# Supplementary material for: Modeling glioblastoma heterogeneity as a dynamic network of cell states
Source: Mol Syst Biol. 2021 Sep 16;17(9):e10105. doi: 10.15252/msb.202010105 (PMC8444284; doi:10.15252/msb.202010105)
Supplement: Supplementary file 6 — Source Data for Figure 5 [file MSB-17-e10105-s004.zip › Figure5A_sourcedata/GSEA_3017/nosigdb_stateB.GseaPreranked.1622795021725/RICHARDS_NOWAKOWSKI_SCIENCE_2017_NEN_EARLY1_UPREG.html]

Details for gene set RICHARDS\_NOWAKOWSKI\_SCIENCE\_2017\_NEN\_EARLY1\_UPREG[GSEA]

|  || Dataset | state43017 |
| Phenotype | NoPhenotypeAvailable |
| Upregulated in class | na\_neg |
| GeneSet | RICHARDS\_NOWAKOWSKI\_SCIENCE\_2017\_NEN\_EARLY1\_UPREG |
| Enrichment Score (ES) | -0.39764524 |
| Normalized Enrichment Score (NES) | -2.7771366 |
| Nominal p-value | 0.0 |
| FDR q-value | 0.0 |
| FWER p-Value | 0.0 |
Table: GSEA Results Summary

  

Fig 1: Enrichment plot: RICHARDS\_NOWAKOWSKI\_SCIENCE\_2017\_NEN\_EARLY1\_UPREG      
 Profile of the Running ES Score & Positions of GeneSet Members on the Rank Ordered List

  

| PROBE | GENE SYMBOL | GENE\_TITLE | RANK IN GENE LIST | RANK METRIC SCORE | RUNNING ES | CORE ENRICHMENT || 1 | CSRP2 |  |  | 96 | 0.539 | -0.0896 | No |
| 2 | PDE1C |  |  | 166 | 0.445 | -0.1494 | No |
| 3 | VRK1 |  |  | 238 | 0.388 | -0.2164 | No |
| 4 | CCSAP |  |  | 249 | 0.379 | -0.2000 | No |
| 5 | CALM2 |  |  | 290 | 0.354 | -0.2269 | No |
| 6 | OLFM1 |  |  | 325 | 0.337 | -0.2470 | No |
| 7 | EFNB2 |  |  | 374 | 0.317 | -0.2880 | No |
| 8 | MID1 |  |  | 375 | 0.317 | -0.2627 | No |
| 9 | SOGA1 |  |  | 378 | 0.314 | -0.2403 | No |
| 10 | ZNF714 |  |  | 382 | 0.313 | -0.2194 | No |
| 11 | EZR |  |  | 403 | 0.307 | -0.2225 | No |
| 12 | CBX5 |  |  | 457 | 0.291 | -0.2725 | No |
| 13 | SEMA3A |  |  | 460 | 0.290 | -0.2521 | No |
| 14 | EML1 |  |  | 515 | 0.278 | -0.3044 | No |
| 15 | KIFAP3 |  |  | 518 | 0.278 | -0.2850 | No |
| 16 | TSPAN13 |  |  | 528 | 0.276 | -0.2754 | No |
| 17 | PDGFC |  |  | 532 | 0.275 | -0.2575 | No |
| 18 | RAI14 |  |  | 572 | 0.269 | -0.2899 | No |
| 19 | PUM2 |  |  | 613 | 0.261 | -0.3243 | No |
| 20 | NRCAM |  |  | 633 | 0.258 | -0.3300 | No |
| 21 | HMGCR |  |  | 683 | -0.257 | -0.3772 | Yes |
| 22 | TUBA1A |  |  | 686 | -0.263 | -0.3590 | Yes |
| 23 | SCD |  |  | 688 | -0.263 | -0.3393 | Yes |
| 24 | GDI2 |  |  | 697 | -0.274 | -0.3285 | Yes |
| 25 | CHD7 |  |  | 706 | -0.284 | -0.3169 | Yes |
| 26 | PRDX1 |  |  | 719 | -0.326 | -0.3074 | Yes |
| 27 | FGFBP3 |  |  | 721 | -0.334 | -0.2821 | Yes |
| 28 | SQLE |  |  | 735 | -0.405 | -0.2677 | Yes |
| 29 | ATCAY |  |  | 738 | -0.417 | -0.2372 | Yes |
| 30 | HMGCS1 |  |  | 739 | -0.418 | -0.2038 | Yes |
| 31 | PPA1 |  |  | 742 | -0.424 | -0.1727 | Yes |
| 32 | GAP43 |  |  | 743 | -0.428 | -0.1385 | Yes |
| 33 | MLLT11 |  |  | 744 | -0.430 | -0.1042 | Yes |
| 34 | TUBB3 |  |  | 747 | -0.448 | -0.0712 | Yes |
| 35 | SNHG3 |  |  | 749 | -0.492 | -0.0332 | Yes |
| 36 | DANCR |  |  | 753 | -0.572 | 0.0083 | Yes |
Table: GSEA details [plain text format]

  

Fig 2: RICHARDS\_NOWAKOWSKI\_SCIENCE\_2017\_NEN\_EARLY1\_UPREG: Random ES distribution      
 Gene set null distribution of ES for **RICHARDS\_NOWAKOWSKI\_SCIENCE\_2017\_NEN\_EARLY1\_UPREG**
